# Analysis of genetic testing in fetuses with congenital heart disease of single atria and/or single ventricle in a Chinese prenatal cohort

**DOI:** 10.1186/s12887-023-04382-7

**Published:** 2023-11-18

**Authors:** Min Li, Baoying Ye, Yiyao Chen, Li Gao, Yi Wu, Weiwei Cheng

**Affiliations:** 1 Prenatal Diagnosis Center, the International Peace Maternity and Child Health Hospital, School of Medicine, Shanghai Jiao Tong University, Shanghai, China; 2 Shanghai Key Laboratory of Embryo Original Diseases, Shanghai, China; 3 Shanghai Municipal Key Clinical Specialty, Shanghai, China; 4 Department of Ultrasonography, the International Peace Maternity and Child Health Hospital, School of Medicine, Shanghai Jiao Tong University, Shanghai, China; 5 Department of Reproductive Genetics, the International Peace Maternity and Child Health Hospital, School of Medicine, Shanghai Jiao Tong University, Shanghai, China

**Keywords:** Congenital heart disease, Chromosomal microarray analysis, Whole exome sequencing

## Abstract

**Objective:**

This study aimed to investigate the genetic etiologies of fetuses with single atria and/or ventricle (SA or/and SV) using different genetic detection methods in a Chinese prenatal cohort.

**Methods:**

In this retrospective study, the various genetic results of 44 fetuses with SA and/or SV were analyzed. All 44 cases were tested by chromosomal microarray analysis (CMA) and karyotyping simultaneously, and 8 underwent whole exome sequencing (WES). Data on the pregnancy outcomes and neonatal prognoses were collected from medical records and postnatal follow-up.

**Results:**

The whole cohort of 44 fetuses included 14 SA cases (31.8%), 12 SV cases (27.3%), and 18 SA and SV cases (40.9%). A total of 9 pathogenic genetic results were detected by conventional karyotyping, CMA and trio-WES, indicating an overall detection rate of 20.5% (9/44). Six pathogenic chromosomal abnormalities were identified by CMA among the 44 cases, showing a detection rate of 13.6% (6/44). Two microdeletions being missed by karyotyping were diagnosed by CMA, showing an additional diagnostic yield of 4.5% for CMA in present cohort(2/44). Three pathogenic variants in two fetuses were identified by WES, indicating an incremental diagnostic yield of 4.5%(2/44) for WES in fetuses with SA or/and SV.

**Conclusion:**

In this study, WES achieved an additional diagnostic yield of 4.5% in fetuses with SA or/and SV. WES is valuable for fetal prognosis assessment and could add diagnostic value for fetuses with SA and/or SV when CMA is negative. It would be a valuable technique for the identification of underlying pathogenic variants in prenatal cohorts.

## Introduction

Congenital heart disease (CHD) is one of the most common birth defects, affecting approximately 1% of neonates in the world [1–4]. Reportedly, approximately 20% of CHDs are caused by pathogenic genetic factors [5]. Single atria and/or single ventricles (SA and/or SV) are complex and severe cardiac malformations, that are associated with very high mortality, and 4–16 in every 100,000 new-borns are affected [6]. However, there have been few prenatal research studies of in fetuses with SA and/or SV, particularly in relation to the genetic etiologies of SA and/or SV in prenatal cohorts.

Our study is the first to conduct comprehensive genetic testing for SA and/or SV in the prenatal period in a Chinese prenatal cohort. The purpose of our study was to explore the genetic etiology of fetuses with SA and/or SV using different genetic testing techniques and to evaluate the clinical application of molecular diagnostic techniques in detecting etiologies of SA and/or SV.

## Materials and methods

### Study population

**Fig. 1:**
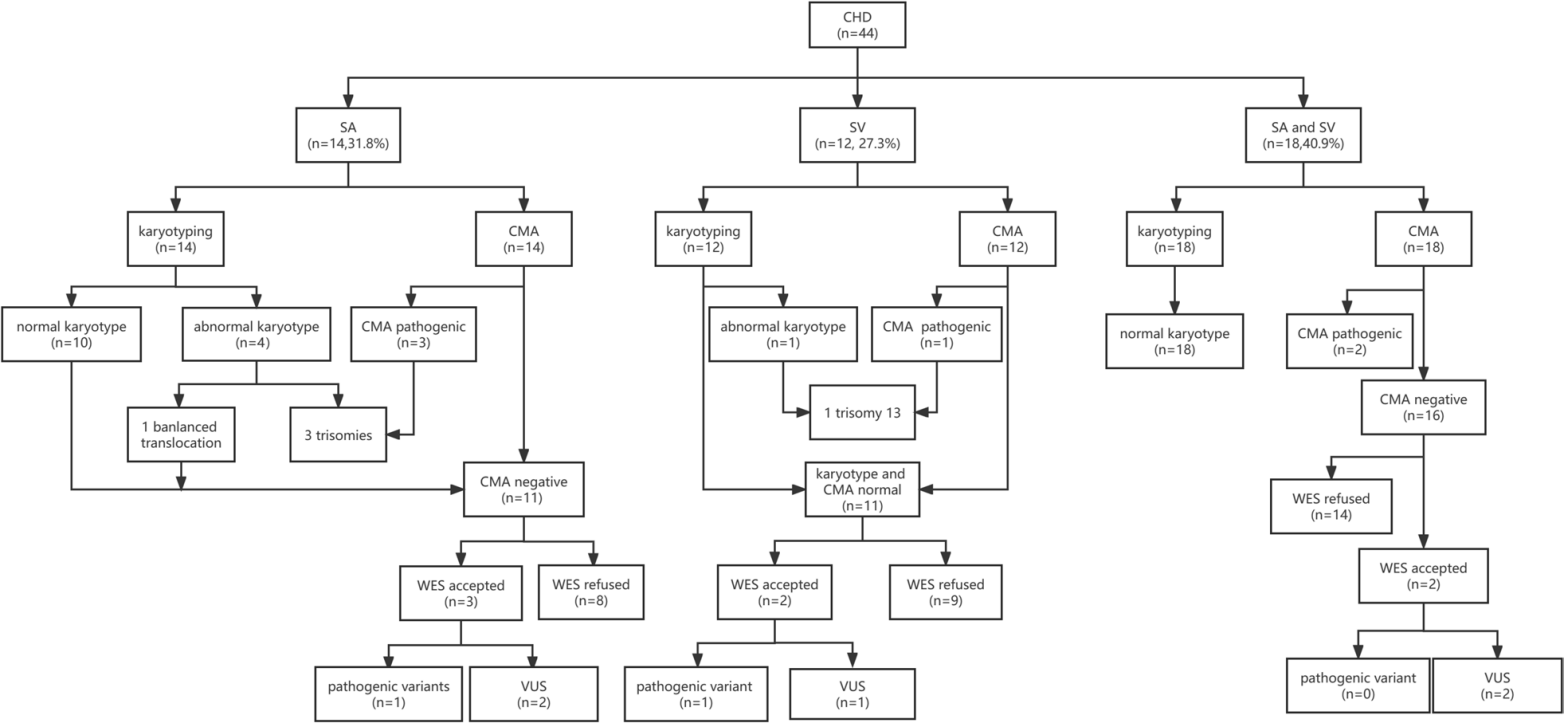
The work-up flow. CHD: Congenital heart disease, SA: single atria, SV: single ventricle, CMA: chromosomal microarray analysis, WES: whole exome sequencing, VUS: variants of uncertain significance

This was a retrospective study. A total of forty-four fetuses with SA and/or SV detected by fetal echocardiology were included in the present study in the International Peace Maternal & Child Health Hospital from Jan 2018 to Dec 2021. The results of fetal echocardiography and genetic testing were reviewed and collected from electronic medical records. Among the 44 cases, 14(31.8%) cases were SA, 12(27.3%) were SV, and the rest (40.9%) were SA combined with SV. After informed consent was obtained, amniocentesis was performed for all 44 patients. CMA was used as the first-line testing to detect the genetic etiologies. Karyotyping was also suggested to identify chromosomal translocations. All 44 pregnant women agreed that CMA and karyotyping were performed simultaneously. For those with negative CMA results and normal karyotypes, whole exome sequencing (WES) was suggested to detect the underlying pathogenicity. The work-up flow chart is shown in Fig. 1.

This study was approved by the Ethics Commission of the International Peace Maternal and Child Health Hospital.

### Fetal echocardiography

The principles of fetal echocardiography cardiac ultrasound were based on the Guidelines of the International Society of Ultrasound in Obstetrics and Gynecology (ISUOG) [7]. Radiologists qualified for prenatal ultrasound diagnosis performed ultrasound examination by GE Voluson E10 (GE Healthcare, Zipf, Austria), Philips iU Elite (Philips, Copenhagen, Denmark), or Philips iE33 (Philips, Copenhagen, Denmark). Each participant exposed her lower abdomen and remained supine on the examination bed. First, it is essential to first determine fetal laterality – identify the right and left sides of the fetus based on its position in utero, before confirming that both the stomach and heart are located on the left side of the fetus. On this basis, the atrioventricular connection relationship was determined by the four-chamber view and veno-atrial connection view. Connection between ventricles and great arteries was determined by the left and right ventricular outflow tract, long axis and short axis view, three-vessel or three-vessel tracheal view, aortic arch view, and ductus arteriosus arch view. The ratios of the left and right atrial diameters, the left and right ventricular diameters, and the aortic arch diameters to the ductus arteriosus diameters were calculated and stored. Ultrasound anatomical diagnosis of fetal cardiac anomalies was finally made by fetal cardiac ultrasound examination by two experienced echocardiologists.

Due to the septum primum and septum secundum being absent during embryonic development, SA has a single atrial cavity. SV occurs when both atrial chambers connect to a single ventricular chamber [8]. The cases of functional single atrium or single ventricle and the CHD types that could potentially develop into functional SA and/or SV (such as hypoplastic left heart syndrome) were not included in this study.

### Chromosomal microarray analysis (CMA) and karyotyping

Invasive procedures and follow-up genetic testing were suggested for all patients. After informed consent was obtained, amniocentesis was performed and amniotic fluid was used for CMA and karyotyping. Genomic DNA was extracted from 5–6 ml amniotic fluid using a Tianamp Micro DNA Kit (Tiangen, Biotech, China), according to the manufacturer’s protocol. DNA was screened with an Affymetrix Cytoscan 750 K (Agilent Santa Clara, Clifornia). The results were analyzed with Affymetrix Chromosome Analysis Suite software (ChAS)0.3.2 (Affymetrix, Santa Clara, California). According to the guidelines of the American College of Medical Genetics and Genomics (ACMG) [9], the detected copy number variations (CNVs) were divided into five different types: pathogenic(P), likely pathogenic (LP), variants of uncertain significance (VUS), likely benign (LB), and benign(B).

Twenty millilitres of amniotic fluid was used for cell culture and karyotyping. After cell culturing, G-banding was performed, and five metaphase cells were examined to detect structural chromosomal abnormalities. At least 15 metaphase chromosomes were examined to detect numerical abnormalities of chromosomes.

### Whole exome sequencing (WES)

Amniocentesis was performed at 17–22 weeks of gestation under continuous ultrasonographic guidance. Forty millilitres of amniotic fluid sample were obtained, twenty millilitres for karyotyping, ten millilitres for CMA, and the remaining ten millilitres for WES. DNA samples obtained from the index family were sequenced using targeted next generation sequencing. Library construction and paired-end sequencing (2 × 100 bp) were performed by Beijing Genomics Institute (BGI) according to the manufacturer’s protocols. Flited clean data were aligned to the human reference genome GRCh37/hg19 by Burrows‒Wheeler Aligner (BWA; v.0.7.12). GATK (version 3.5) and ANNOVAR [10] were used for variant calling and annotation, respectively. The interpretation of variants was based on the ACMG/AMP guidelines [11].

## Results

### Diagnostic yield of CMA

In the present study, a total of 9 pathogenic genetic results were detected by conventional karyotyping, CMA and trio-WES, indicating an overall detection rate of 20.5% (9/44, seen in Table 1). Most of the abnormal genetic results were found in the SA group (5/44, 11.4%). CMA identified six cases with pathogenic copy number variations (CNVs), indicating a CMA detection yield of 13.6% (6/44, seen in Table 2). Conventional karyotyping identified five cases with chromosomal abnormalities, including four aneuploidies and one chromosomal translocation (seen in Table 3). Four aneuploidies were also detected by CMA. Regarding to the case with balanced translocation (case 5 in Table 3), no pathogenic findings were detected by CMA and the subsequent trio-WES. Hence, this balanced translocation was not considered CHD causative. Two pathogenic microdeletions that were missed by conventional karyotyping were identified by CMA, showing an additional diagnostic yield of 4.5% (2/44). These two pathogenic CNVs were 16p12.2 deletion and 11q24.1q24.3 deletion (cases 5 and 6 in Table 2). Among the 6 pathogenic genetic results detected by CMA and karyotyping, chromosomal numerical abnormalities accounted for 66.7% of the pathogenic cases (4/6), with the most common aneuploidy being trisomy 18.

**Table 1:** Genetic testing results in three groups

Classification	Number of fetuses	genetic testing	
		Abnormal	normal
SA	14(31.8%)	5(11.4%)	9(20.5%)
SV	12(27.3%)	2(4.5%)	10(22.7%)
SA and SV	18(40.9%)	2(4.5%)	16(36.4%)
Total	44(100.0%)	9(20.5%)	35(79.5%)

*SA* Single atria, *SV* Single ventricle

**Table 2:** Pathogenic results detected in CMA

Case	MA	GA	Clinical CHD phenotypes	Genetic results	Pregnancy outcome
1	35	23^+3^	SA, DORV, PA, AS	Trisomy 18	TP
2	36	22^+6^	SA	Trisomy 21	TP
3	43	20^+1^	SA, HLHS	Trisomy 18	TP
4	31	29^+3^	SV, GVD, AVD	Trisomy 13	TP
5	29	22^+1^	SA + SV, PTA	arr[hg19]16p12.2(21,740,199–22,442,007) × 1	TP
6	26	23^+5^	SA + SV, PTA	arr[hg19]11q24.1q24.3(121,235,870–130,733,365) × 1	TP

*SA* Single atria, *SV* Single ventricle, *MA* Mean maternal age, *GA* Gestational age, *TP* Termination of pregnancy, *DORV* Double-outlet right ventricle, *PA* Pulmonary atresia, *AS* Aortic stenosis, *HLHS* Hypoplastic left heart syndrome, *GVD* Great vessel dysplasia, *AVD* Atrioventricular valve dysplasia, *PTA* Persistent truncus arteriosus

**Table 3:** Abnormal results of karyotyping in three groups

Case	MA	GA	Karyotype	Clinical CHD phenotypes	Postnatal outcome
1	35	23^+3^	Trisomy 18	SA, DORV, PA, AS	TP
2	36	22^+6^	Trisomy 21	SA	TP
3	43	20^+1^	Trisomy 18	SA, HLHS	TP
4	31	29^+3^	Trisomy 13	SV, GVD, AVD	TP
5	22	25	46,XN,t(4; 11) (p16.3; p13)	SA	TP

*SA* Single atria, *SV* Single ventricle, *MA* Mean maternal age, *GA* Gestational age, *TP* Termination of pregnancy, *DORV* Double-outlet right ventricle, *PA* Pulmonary atresia, *AS* Aortic stenosis, *HLHS* Hypoplastic left heart syndrome, *GVD* Great vessel dysplasia, *GVD* Great vessel dysplasia, *AVD* Atrioventricular valve dysplasia

### Diagnostic yield of WES

For the remaining 38 cases with normal karyotypes and negative CMA results, trio-WES was suggested to detect the underlying pathogenicity. However, further genetic testing by trio-WES was accepted by only eight participants. In one case, CMA and trio-WES were performed in parallel to obtain a quick round-around time. The patient’s CMA and WES results both indicated the pathogenic microdeletion of 16p12.2, which could lead to abnormal fetal heart development. This case was not accounted for when calculating the additional diagnostic yield of WES.

Three pathogenic variants in two fetuses were revealed by trio-WES, namely *HPGD* and *EVC2*. In one fetus with SA, the WES results revealed an autosomal recessive inheritance mode of *EVC2*. The complex heterozygous mutation of *EVC2* which was inherited from the father and mother, led to Ellis-van Creveld Syndrome. Excluding the case of 16p12 microdeletion which was simultaneously detected by CMA, we found that trio-WES obtained an incremental diagnostic yield of 4.5% (2/44), with 50% in the SA group (1/2) and 50.0% in the SV group (1/2). In addition to the three pathogenic variants, five VUS variants were observed. The WES results of the eight cases are shown in Table 4.

**Table 4:** The results of exome sequencing in seven fetuses with SA and(or) SV

Case	MA	GA	Clinical CHD phenotypes	Structural anomalies other than CHD	Genetic results	Nature	inheritance	Outcome
1	29	23^+1^	SA, double outlet of right ventricle	/	NM_002816.3(PSMD12):c.347C > T(p.Thr116Ile)	VUS	Maternal	TP
2	27	24^+3^	SA	short limbs and narrow thorax	NM_147127.4(EVC2):c.1195C > T(p.Arg399*) NM_147127.4(EVC2):c.1987G > T(p.Glu663*)	P	Paternal Maternal	TP
3	31	22^+4^	SA, double outlet of right ventricle, VSD	absence of spleen	NM_003482.3(KMT2D):c.2264G > C(p.Arg755Pro)	VUS	Unknown	TP
4	31	23^+2^	SV, transposition of great arteries	heterotaxy	NM_003482.3(KMT2D):c.16351 T > C(p.Tyr5451His)	VUS	Paternal	TP
5	31	19^+5^	SV, permanent arterial trunk	/	NM_000860.5(HPGD):c.310_311delCT(p.Leu104Alafs*3)	P	De novo	TP
6	30	19^+6^	SA + SV	/	NM_007373.3(SHOC2):c.1273G > A(p.Val425Ile) NM_001039590.2(USP9X):c.5844 T > C(p.Ala1948Ala) NM_014008.3(CCDC22):c.1820G > A(p.Arg607Gln)	VUS	Maternal Maternal Paternal	TP
7	30	23^+1^	SA + SV,permanent arterial trunk	/	NM_016008.3(DYNC2LI1):c.574C > G(p.Gln192Glu) NM_015662.1(IFT172):c.918A > G(p.Leu306Leu) NM_015662.1(IFT172):c.3052C > T(p.Arg1018Cys)	VUS	Maternal Maternal Paternal	TP
8	29	22^+1^	SA + SV	/	arr[hg19]16p12.2(21,740,199–22,442,007) × 1	P	De novo	TP

*SA* Single atria, *SV* Single ventricle, *MA* Mean maternal age, *GA* Gestational age, *TP* Termination of pregnancy

### Pregnancy outcome

Most parents (90.9%, 40/44) decided to terminate the pregnancy as soon as possible, and in only 4 (9.1%, 4/44) patients had live births by caesarean section. The gestational week at birth, neonatal weight and neonatal outcomes are shown in Table 5. Two fetuses with a prenatal ultrasound diagnosis of SA underwent stage I surgery one week after birth, and these two children had survived to one year of age by the time of follow-up. The parents indicated that their children's physical development and immunity were relatively weak compared to those of children of the same age and that their children were more prone to viral infection and illness. Another two fetuses with prenatal ultrasound diagnoses of SA and SV also underwent Stage I surgery after birth, but unfortunately, both died within a month after the operation.

**Table 5:** Pregnancy outcomes of the four fetuses in which pregnancy was continued

Number	Prenatal CHD phenotypes	Genetic testing (karyotype + CMA + WES)	Gestational weeks at birth	Delivery mode	Gender	Birth Weight(g)	Surgery	Outcomes
1	SA	Normal	34^+5^	CS	M	2080	Stage I	One year old, height 63 cm weight 6.9 kg, prone to illness
2	SA	Normal	36^+2^	CS	F	2460	Stage I	One year old, height 65 cm weight 6.9 kg, prone to illness
3	SA + SV	Normal	37^+5^	CS	M	2310	Stage I	Died one month after surgery
4	SA + SV	Normal	39^+1^	CS	M	2970	Stage I	Died two weeks after surgery

*SA* Single atria, *SV* Single ventricle, *CS* Caesarean section, *M* Male, *F* Female

Autopsy to obtain more detailed phenotypic information was recommended to all parents who decided to terminate their pregnancies. However, few parents accepted it due to their secular views. Only four couples chose fetal autopsy. After informed consent was obtained, fetal autopsies were performed at the Department of Pathology, the International Peace Maternity & Child Health Hospital. The autopsy findings of fetal specimens were carefully reported by specialized pathologists.

Three cases, two female fetuses and one male fetus, were from the SA group. The autopsy of one female fetus showed consistent findings with prenatal ultrasound of a single atrium, transposition of the great arteries and an intact ventricular septum. Another female fetus was found to have complex heart disease involving a single atrium, pulmonary atresia and ventricular septal defect. In addition to the cardiac malformations, autopsy revealed the absence of the spleen. Autopsy of the male fetus showed micro-limb deformity (short humerus and femur), polydactyly (six fingers on both hands with wide palms) and atrioventricular septal defect. One patient in the SA and SV groups was male, and his autopsy report was complex cardiac malformations – dextrocardia, SA, SV, three lobes of left lung, and absence of spleen. Fetal cardiac autopsy findings were in accordance with ultrasound diagnoses.

## Discussion

In the present study, the overall detection rate for SA and/or SV was 20.5% (9/44), including chromosomal abnormalities and pathogenic genetic variants. Our results were lower than those of Wang et al. 's study [12], which reported that approximately one-third of patients with SV had pathogenic chromosomal aberrations. This might be caused by selection bias and the different technical platforms used in the two groups. The study population in Wang's group was postnatal children, including various kinds of SV types of HLHS (hypoplastic left heart syndrome), DORV (double outlet right ventricle), etc. Our study was focused mainly on prenatal fetuses whose fetal echocardiography showed single atrium and/or single ventricles, not including the CHD types that could potentially develop into functional SA and/or SV (e.g., HLHS). The incremental detection rate of trio-WES for SA and/or SV in the present study was noted as 4.5% (2/44), much lower than that in Xing et al. 's study [13] (4.5% vs 12.8%), which was mainly due to the small sample size. In our study, only eight mothers underwent trio-WES to detect the underlying pathogenic variants. If all patients accepted trio-WES, the incremental diagnostic yield of prenatal WES would be much higher than 4.5%.

The overall detection rate of CMA in our study was 13.6% (6/44), which was similar to previous studies with diagnostic yields of CMA ranging from 11 to 21% [12–14]. The most common numerical chromosomal abnormalities in the present study were trisomy 18 in 2 cases. This is consistent with Xing et al. 's report [13] that trisomy 18 was the most common aneuploidy in CHD fetuses. According to previous studies [15–17], the most common submicroscopic genetic aberration in CHD is 22q11.2 deletion, which might account for approximately 5% of CHD patients. In this study, conotruncal defects were observed in 75.0% (33/44) of SA/SV fetuses, while 25.0% (11/44) did not exhibit such defects. However, the 22q11.2 deletion was not observed in fetuses with SA and/or SV, the main reason might be due to the small sample size.. Most likely for the same reasons, our results indicated an incremental diagnostic yield of 4.5% on CMA (4.5%, 2/44) which was lower than the previously reported incremental diagnostic yield of CMA ranging from 8 to 11% in CHD [18–20]. Whether the incidence of 22q11.2 deletion is relatively low in the special population of SA and/or SV still requires large-scale research to confirm.

The 16p12.2 microdeletion (case 5 in Table 2, case 8 in Table 4), which was detected by both CMA and WES, is a roughly 520-kb deletion with varied expressivity and partial penetrance. This genetic region contains several OMIM genes including *OTOA*, *UQCRC2*, and *EEF2K*. 16p12.2 microdeletion has been linked to cleft lip and neurodevelopmental problems [21]. It is also associated with cardiac defects. Linnane et al. reported a patient with 16p12.2 microdeletion presenting tetralogy of Fallot with an absent pulmonary valve [22]. We also found several patients in the DECIPHER database who carried deletions very similar to our patient. These patients exhibited various heart malformations, such as tetralogy of Fallot, abnormal heart morphology and ventricle septal defect(patients' number in DECIPHER database: 250,875,1579,261,540). The deletion of 11q24.1q24.3 detected in another fetus (case 6 in Table 2) spans approximately 9.5 Mb (chr11: 121,235,870–130,733,365), including a large number of OMIM genes such as *DCPS*, *CDON*, *FLI1*, etc. The deletion of this genetic segment may lead to multiple organ abnormalities, such as brain malformation, kidney dysplasia, congenital heart disease and so on. Several cases with similar 11q24.1q24.3 deletions in DECIPHER database showed different cardiac anomalies, such as atrial septal defect and ventricle septal defect(patient number in DECIPHER database: 278131278,596,282,318).

The pathogenic variations in *HPGD* cause primary hypertrophic osteoarthropathy (PHO), inherited in an autosomal recessive mode. The main clinical symptoms of PHO include skin thickening and excessive sweating, delayed closure of the cranial sutures and congenital heart disease, especially patent ductus arteriosus [23]. However, we identified only a variant in *HPGD* gene in this patient, and did not find another possible SNV or CNV (case 5 in Table 4). This variant was pathogenic, but for this fetus, this variant was not CHD causative.

One fetus presenting with an endocardial cushion defect and a single atrium in the current study was found to carry a compound heterozygous mutation of *EVC2* by WES which would cause a rare genetic disease, namely, Ellis-van Creveld syndrome [24, 25] (case 2 in Table 4). Ellis-van Creveld syndrome is an autosomal recessive genetic disease. Its main clinical symptoms are chondrodystrophy, polydactyly, ectoderm dysplasia triad and cardiac malformation. The patient would also show signs including short leg dwarfism, short ribs, multiple fingers behind the axis, and dysplasia of nails and teeth, among others. Approximately 60% of patients with Ellis-van Creveld syndrome also show congenital heart defects, the most common of which are atrioventricular septal defects and a single atrium [26]. We reviewed the patient's electronic records, which showed that the mother had an adverse pregnancy two years ago in which she had a fetus with very short long bones and cardiac phenotype of atrioventricular septal defect. However, she refused genetic testing at that time and terminated the pregnancy. Considering the two adverse pregnancies, we thought that the parents were most likely to be carriers of pathogenic *EVC2* variants. The parents finally accepted the suggestion of performing family validation using Sanger sequencing. The results of family validation revealed that the pathogenic *EVC2* variant c.1195C > T (p.Arg399*) was inherited from the father, and the likely pathogenic *EVC2* variant of 1987G > T (p.Glu663*) was inherited from the mother. Hence, according to Mendelian inheritance, the couple would have a high one-fourth risk of having a fetus with Ellis-van Creveld Syndrome if they chose to conceive naturally next time. PGT-M was strongly suggested to avoid repeated adverse pregnancy. This case shows the great value of prenatal WES in defining the risk of recurrence and instructing the mode of the next pregnancy to avoid repeated adverse pregnancy.

## Conclusion

In summary, our data support the view that trisomy 18 is the most common aneuploidy in CHD fetuses, especially in SA. Our study also demonstrated that invasive procedures with CMA should be offered for fetuses with SA and/or SV as first-line genetic testing to determine whether there are genetic abnormalities, as CMA achieved a detection rate of 13.8% (6/44). Only eight participants accepted WES for further genetic testing, and among those who did, WES identified five VUS and three pathogenic variants, indicating an incremental diagnostic of 4.5%. The incremental diagnostic yield of WES is valuable for subsequent clinical management of fetuses in SA and/or SV with normal karyotype and negative CMA results.

## Strength and limitations

To our knowledge, this is the first study to evaluate the results of genetic testing using CMA and WES prenatally in fetuses with SA and/or SV. This is also the first study to summarize the pregnancy outcomes of fetal SA and/or SV cases, providing useful information for prenatal counselling and pregnancy management.

The main limitation is the small sample size of this study, which would lead to result deviation and incorrect statistical results. Another limitation is that not all participants accepted WES, due to the high cost and lack of awareness of genetic abnormalities. Further large-sample multicentre studies are warranted to evaluate the prenatal diagnosis of SA and/or SV.

## Data Availability

All novel variants have been submitted to the NCBI ClinVar database whose accession number is SCV004037445—SCV004037447 (https://www.ncbi.nlm.nih.gov/clinvar/submitters/509288/).
